# The Effectiveness of High-Frequency Repetitive Transcranial Magnetic Stimulation on Patients with Neuropathic Orofacial Pain: A Systematic Review of Randomized Controlled Trials

**DOI:** 10.1155/2022/6131696

**Published:** 2022-08-24

**Authors:** Yingxiu Diao, Yuhua Xie, Jiaxin Pan, Manxia Liao, Hao Liu, Linrong Liao

**Affiliations:** ^1^Rehabilitation Medicine Center, The First Dongguan Affiliated Hospital, Guangdong Medical University, Dongguan, Guangdong 523710, China; ^2^School of Rehabilitation Medicine, Gannan Medical University, Ganzhou, Jiangxi 341000, China; ^3^Department of Rehabilitation, Yixing JORU Rehabilitation Hospital, Wuxi, Jiangsu 214200, China

## Abstract

**Background:**

Repetitive transcranial magnetic stimulation (rTMS) has been widely used in the treatment of neuropathic orofacial pain (NOP). The consistency of its therapeutic efficacy with the optimal protocol is highly debatable.

**Objective:**

To assess the effectiveness of rTMS on pain intensity, psychological conditions, and quality of life (QOL) in individuals with NOP based on randomized controlled trials (RCTs).

**Methods:**

We carefully screened and browsed 5 medical databases from inception to January 1, 2022. The study will be included that use of rTMS as the intervention for patients with NOP. Two researchers independently completed record retrieval, data processing, and evaluation of methodological quality. Quality and evidence were assessed using the PEDro scores and the Grading of Recommendations Assessment, Development, and Evaluation (GRADE) system.

**Results:**

Six RCTs with 214 participants were included in this systematic review: 2 studies were considered level 1 evidence, and 4 were considered level 2 evidence. Six studies found that high-frequency rTMS had a pain-relieving effect, while 4 studies found no improvement in psychological conditions and QOL. Quality of evidence (GRADE system) ranged from moderate to high. No significant side effects were found.

**Conclusions:**

There is moderate-to-high evidence to prove that high-frequency rTMS is effective in reducing pain in individuals with NOP, but it has no significant positive effect on psychological conditions and QOL. High-frequency rTMS can be used as an alternative treatment for pain in individuals with NOP, but further studies will be conducted to unify treatment parameters, and the sample size will be expanded to explore its influence on psychological conditions and QOL.

## 1. Introduction

Neuropathic orofacial pain (NOP) is a specific neurological disorder, usually caused by the somatosensory nervous system or related disorders [1]. NOP mainly affects women over the age of 50 years old [2]. The prevalence varies from 0.03% to 0.5%, depending on the type and characteristics of the disease [3, 4]. The diagnosis of NOP requires the history of peripheral nervous system injury and the distribution of neuroanatomy pain. This disease exists in several specific forms, including pathologies such as atypical facial pain (AFP), burning mouth syndrome (BMS) (also known as glossodynia), trigeminal neuralgia (TN), persistent idiopathic facial pain (PIFP), and postherpetic neuralgia (PHN) [5]. NOP belongs to chronic peripheral neuropathic pain, involving a variety of neurotransmitters and mechanisms. Pain conditions in the mouth and face can interfere with activities of daily living and interfere with communication, eating, and other pleasures of social life, which can result in patients being isolated by society [6]. This kind of disease is relatively common in life, and it not only brings a great economic burden to the patient's family but also affects the patient's physical and psychological health. NOP can be treated with minimally invasive therapy [7], surgery [8], and adjuvant analgesics [9], but people tend to be more receptive to treatments that are noninvasive and nondrug dependent [10]. In the vast majority of cases, neuropathic pain is not satisfactorily treated with traditional analgesics and is often resistant to opioids, so most patients are reluctant to receive such treatment [11].

With the development of high technology and highly evidence-based medicine, noninvasive and painless neuromodulation techniques have received more attention and research in recent years. Repetitive transcranial magnetic stimulation (rTMS) is a noninvasive neuromodulation technique that delivers focal stimulation to an individual's brain using locally pulsed magnetic fields [12, 13]. rTMS has various output forms and stimulation modes, each with different characteristics and applications [14, 15]. Theta burst stimulation (TBS), as a treatment mode of rTMS, has also been widely used in the treatment of NOP [16–18]. There are many subtypes of NOP, and pain and other uncomfortable symptoms are mainly confined around the mouth and face. rTMS produces analgesic effects through different mechanisms in the treatment [19–22]. Due to the diversity and complexity of NOP patients, the efficacy of rTMS for NOP is still controversial [13, 23, 24] because the stimulation site, frequency, intensity, and course of rTMS treatment of NOP are not standardized and unified. According to previous studies [18, 25], NOP is the disease that benefits most from rTMS treatment in the motor cortex. Although there have been reviews evaluating the efficacy of rTMS for NOP patients, there have been no comprehensive evaluations specifically for NOP.

Since rTMS has been widely used to treat different types of pain, it is urgent to explore whether rTMS is safe and effective in patients with NOP [26]. Therefore, the primary objective of this systematic review was to evaluate the effectiveness of rTMS on pain intensity, psychological conditions, and QOL in individuals with NOP. The secondary objective was to review the selection of rTMS parameters for patients with different types of NOP.

## 2. Materials and Methods

### 2.1. Study Design and Registration

This research was performed according to the *Cochrane Handbook* [27]. The protocol for this systematic review was registered at the PROSPERO (CRD42021254738). This systematic review is based on RCTs to measure the effectiveness of rTMS in the treatment of NOP symptoms. The participant, intervention, comparison, and outcome (PICO) [28, 29] principle was adopted in this research. It follows the preferred reporting items for systematic reviews and meta-analyses (PRISMA) [30].

### 2.2. Research Question

In this systematic review, patients were divided into the real stimulation group and sham stimulation group, and different parameters of rTMS were used. The physical manifestations of patients were compared. Therefore, this systematic review mainly answers the following two questions. Is rTMS in the experimental group more effective than that in the sham stimulation group in improving the pain, psychological conditions, and QOL of patients with NOP? Are there differences in the selection of rTMS parameters and treatment sites among NOP patients with different symptoms?

### 2.3. Search Strategy

Two researchers (YXD and YHX) independently conducted database searches in PubMed, Embase, Web of Science, Physiotherapy Evidence Database (PEDro), and Cochrane Library to review the titles and abstracts of the retrieved articles to identify articles that meet the criteria and to qualify studies of rTMS in the treatment of NOP from database establishment to publication on January 1, 2022. English language restrictions were applied, and the search terms for each database were slightly modified. Combined medical terms were searched as follows: (“Transcranial Magnetic Stimulation” OR “TMS”) AND (“Neuropathic orofacial pain” OR “Facial pain” OR “Face pain” OR “Trigeminal Neuralgia” OR “Burning mouth syndrome” OR “Persistent idiopathic facial pain” OR “postherpetic neuralgia”).

### 2.4. Study Selection

The entire screening process was completed by two reviewers (YXD and YHX). Eligible articles were published in English. Only RCTs of intervening NOP with rTMS were included in this systematic review. In addition, references to related articles were reviewed to facilitate the search for other research studies. All RCTs involved rTMS for patients with different types of NOP: BMS, PIFP, AFP, TN, and PHN. Interventions included rTMS, and control groups included sham rTMS interventions or placebo controls. All existing disagreements were discussed through a meeting, and then a consensus was reached. The effects of rTMS on pain intensity, QOL, and psychological conditions in individuals with NOP were evaluated using the following outcome indicators. The Visual Analogue Scale (VAS) is primarily used to measure pain intensity [31]. Psychological conditions were assessed by the Self-rating Depression Scale (SDS), Beck Anxiety Inventory (BAI), Beck Depression Inventory (BDI), and other scales, and QOL was assessed by sleep quality (SQ), 36-Item Short-Form Health Survey (SF-36), and other tools.

Exclusion criteria were as follows: (1) rTMS in combination with other interventions; (2) incomplete data or inability to obtain full-text literature; (3) conference reviews, meta-analyses, letters, or case reports; (4) animal experiments; and (5) non-English literature.

### 2.5. Data Extraction

The first author (YXD) analyzed and summarized the characteristics and effects of rTMS on NOP, and then two co-authors (YXD and YHX) further evaluated the accuracy of the extracted data. Differences between the two will be settled by one of the corresponding authors (HL). Relevant information was extracted for each study as follows: article title, authors' names, publication date, number of participants, type of treatment, mean age of participants, inclusion and exclusion criteria of participants, outcome measures, adverse events, study findings, and conclusions.

### 2.6. Risk-of-Bias Assessment in Included Studies

The selected study was rigorously evaluated using the PEDro scale (PEDro scores of 0–3, 4–5, 6–8, and 9–10 were considered to indicate “poor,” “fair,” “good,” and “excellent” quality) [32]. The PEDro scale provides a more scientific measure of methodological quality. Studies using a PEDro rating of good or excellent with a sample size of >50 are considered level 1 evidence, and studies of lower quality are considered level 2 evidence (fair or poor by PEDro with a sample size of ≤50). The quality of the evidence was assessed by using the GRADE system [33], which uses the domains of study limitations, indirectness, inconsistency, and imprecision in results, and was assessed as “high,” “moderate,” “low,” or “very low” [34]. The recommendation strength is divided into two levels: strong recommendation and weak recommendation, and symbols for description are provided. It identified five categories of problems affecting the quality of evidence, including risk of bias, inaccuracy, inconsistency, indirectness, and publication bias.

### 2.7. Data Synthesis and Analysis

The data and characteristics of rTMS on NOP were summarized by the first author (YXD), and the accuracy of the included data was checked by the next two authors (YHX and MXL). Disagreements were resolved by discussion with the principal investigator (LRL) until a consensus was reached. Data on other indicators were also summarized to describe the effects of the intervention. We summarized the relevant data characteristics of rTMS, such as parameters of different frequencies, intensities, and pulse times, as well as different stimulation sites and parameter selection, and summarized the characteristics of rTMS on NOP patients. In this systematic review, patients with BMS, PIFP, AFP, TN, and PHN of different amounts were summarized as NOP, and the effectiveness of rTMS was explored by integrating rTMS data and stimulating sites in patients with different types of NOP. Ultimately, no meta-analysis was performed because the heterogeneity of the studies was too great. Therefore, we present the summarized data as a systematic review.

## 3. Results

### 3.1. Study Selection

**Figure 1:**
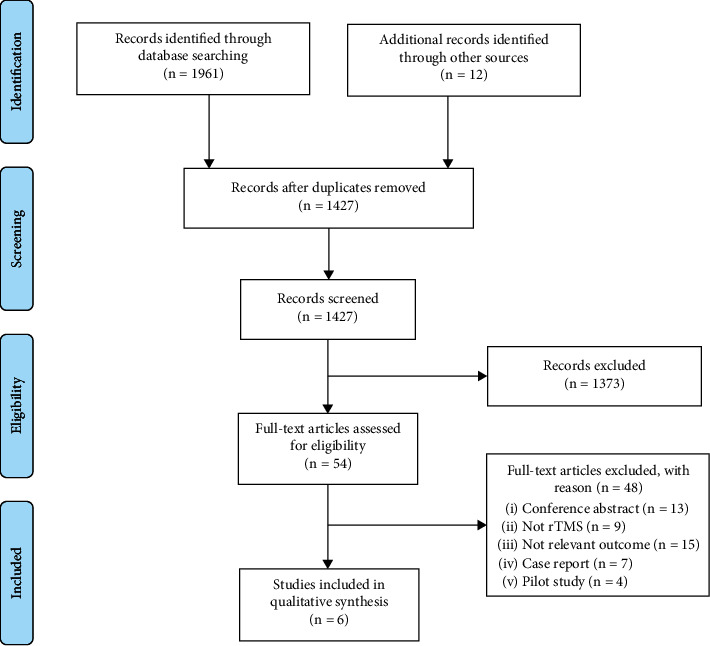
PRISMA flow diagram.

The retrieval strategy identified a total of 1973 articles and 12 additional records identified through other sources. There were still 1427 articles left after removing duplicate items, and 1373 studies were excluded after screening of study titles and abstracts for the following reasons: evaluation of symptoms involving different pathologies and populations different from NOP, systematic reviews, conference abstracts, non-English literature, animal studies, and publications where full texts and data are not available. Subsequently, by full content screening out of 54 RCTs, after assessing their eligibility, 6 RCTs remained.  Figure 1 shows a more detailed description of the literature screening process using the PRISMA flow chart, and six RCTs [18, 25, 35–38] were considered eligible for inclusion in this systematic review.

### 3.2. Methodological Quality Assessment

We could search the Physiotherapy Evidence Database website for PEDro scores included in the studies, but we found that none were registered on it. Thus, this systematic review was reviewed and scored independently by two researchers (YXD and JXP). The differences between the two were resolved by another researcher (HL). Ultimately, six trials reported concealed allocation: three of the studies [18, 25, 35] were double-blind, two studies [36, 38] were single-blind, and the other study [37] did not specify what type of blindness was used (Table 1). Overall, only two studies [25, 35] were considered level 1 evidence (PEDro score ≥ 6 and sample size > 50) and four [18, 36–38] were considered level 2 evidence due to differences in sample sizes. The grading of evidence for each study is summarized in Table 2, and all studies were rated as medium-to-high quality.

### 3.3. Study Characteristics

After strict literature screening and scientific quality evaluation processing, 6 RCTs [18, 25, 35–38] were chosen for this systematic review and these studies were published between 2013 and 2019. The characteristics among which patients have NOP are summarized in Table 3, and the main characteristics of rTMS are summarized in Table 4. Three studies [18, 35, 36] of patients with multiple types of NOP were included. Due to the complexity of the patients, they were uniformly summarized as NOP patients. There were two studies in patients with PHN [25, 37] and one in patients with BMS [38]. Their pain lasts for at least six months and up to 30 years. The coil types used in the rTMS were the figure-of-eight coil [36, 38] and round coil [37]. All included rTMS were high-frequency stimulation, and other related parameters are obtained, such as the intertrain interval (ITI), motor threshold (MT), and total number of pulses. The systematic review provided data on 214 patients who were followed multiple times by evaluators. All the patients included had problems of varying degrees, such as pain, depression, and sleep disorders.

### 3.4. Participants

A total of 214 NOP patients were included in the 6 studies [18, 25, 35–38]. Since the diagnosis types of patients in the 3 studies were not clear [18, 35, 36], the number of each category of NOP patients could not be determined. Ninety-four patients were enrolled in the three studies with orofacial pain duration ranging from one month to 30 years [18, 35, 36]. 100 patients were diagnosed with PHN [25, 37]: 60 in the experimental group and 40 in the nonintervening group, and all patients with PHN had pain lasting more than six months. Among the 16 patients included in the study of Shamseer et al. [28], 4 patients had AFP, 5 patients had BMS, and 7 patients had TN. Most of the patients suffered from intractable pharmacotherapy pain with an average period of pain exceeding six months. The specific number of male and female patients in the experimental group and control group could not be determined, but there were 14 female patients and 2 male patients. Since precise data on the number of gender in the nonintervening group could not be obtained from the study of Cumpston et al. [27], it was impossible to judge the ratio of men and women in the control group. Overall, there were significantly more women in the study than men.

### 3.5. Interventions

In six studies, rTMS targeted the primary motor cortex (M1) [18, 25, 37], left dorsolateral prefrontal cortex (DLPFC) [38], primary sensory cortex (S1) [36], and secondary somatosensory cortex (S2) [36] in patients with NOP. Six studies included five [25, 35–38] high-frequency (5-20 Hz) rTMS treatments and one TBS [18] treatment at 50 Hz, and the control group used sham stimulation or rTMS at different frequencies. Fricová et al. [35] treated NOP patients with 20 Hz and 10 Hz rTMS by stimulating the body part of the contralateral motor cortex that corresponds to the location of the pain and comparing the two parameters at different frequencies to see if symptoms improved. It received five sessions (applications) continuously during working days (days 1-5). Lindholm et al. [36] used a frequency of 10 Hz to intervene in the three targets of patients (M1, S1, and S2) to observe which one could alleviate chronic drug-resistant NOP to the greatest extent and concluded that the right S2 is a promising new target. The stimulation was given in trains of 50 pulses at 10-second intervals and a 15-minute break. The same rTMS parameters were used for PHN in both studies [25, 37] with a frequency of 10 Hz, 80% MT, and a total of 1500 pulses. The duration of each stimulus was 0.5 seconds, and the interval between two stimuli was 3 seconds. rTMS was performed once a day for 15 consecutive days. Frequency selection in six studies [18, 25, 35–38] ranged from 5 Hz to 50 Hz, and the intensity of stimulation ranges from 80% resting motor threshold to 110% resting motor threshold with total pulses ranging from 600 to 3000.

### 3.6. Outcome Measures

The outcome measurements for each RCT are shown in Table 4. The outcomes obtained in this systematic review mainly included three aspects: pain, psychological conditions, and QOL. In all studies, pain was measured by VAS [18, 25, 35, 37, 38], Numerical Rating Scales (NRS) [36], Short-Form McGill Pain Questionnaire (SF-MPQ) [25, 37, 38], and Brief Pain Inventory (BPI) [36, 38]. The assessment of psychological conditions includes BDI [36, 38], Patients' Global Impression of Change (PGIC) [25, 37, 38], SDS [25, 37], Patient Health Questionnaire (PHQ-9) [38], Clinical Global Impression for global improvement scale (CGI-I) [38], and BAI [18]. QOL was assessed by SF-36 [36], BPI [36, 38], neuropathic pain impact on quality of life (NePIQoL) questionnaire [25, 36, 37], and SQ [25, 37].

### 3.7. Effectiveness

#### 3.7.1. Effect of rTMS on Pain Intensity

The effectiveness of rTMS protocols in patients with NOP is summarized in Table 4. The pain intensity of NOP was evaluated using VAS and NRS in these studies [18, 25, 35–38], and 214 patients were involved in the rTMS group and control groups. Five studies [25, 35–38] have shown that rTMS can significantly improve pain in patients with NOP, but in another study [18], iTBS of M1 can temporarily relieve temporary and moderate subjective pain in NOP patients. In Fricová et al. [35], we found about a 24% reduction in pain values in patients with NOP. In Lindholm et al.'s [36] study, we found a 38% reduction in pain. Umezaki et al. [38] found that patients in the real group reported a 67% decrease in BMS pain intensity, and 75% of the subjects in the real group reported a >50% decrease in BMS pain intensity from baseline to day 60. We found that in Kohútová et al.'s [18] study, patients' pain values decreased by about 15%. Pei et al. [25] found that the pain value of PHN patients decreased by 39.89%. Ma et al. [37] found that the mean VAS reduction in the real rTMS group was 16.89% for the duration of disease longer than 6 months. In summary, all six studies showed that high-frequency rTMS had a positive effect on reducing pain.

#### 3.7.2. Effect of rTMS on Psychological Conditions

Major indicators such as SDS and BDI were used to evaluate the psychological status of patients. Five studies [18, 25, 36–38] analyzed the psychological conditions of patients, but compared with the nonintervening group, patients showed no significant changes in anxiety and depression. Therefore, the above results indicate that rTMS has no obvious advantage in improving patients' psychological conditions, especially anxiety and depression.

#### 3.7.3. Effect of rTMS on Quality of Life

Four studies [25, 36–38] reported on patients' QOL, sleep quality, and so on. Measures of QOL will be combined with patient activities and participation, but there was a slight reduction in the QOL score. In addition, Lindholm et al. [36] showed a slight improvement in patients' QOL, while the other four studies did not have a positive impact on the improvement of QOL, including sleep quality and patients' physical health. Therefore, rTMS does not significantly improve QOL in individuals with NOP.

### 3.8. Adverse Effects

Minor adverse events associated with rTMS treatment were reported in five studies [18, 25, 36–38], and only one study reported no significant adverse events. Umezaki et al. [38] found that patients had side effects of headache symptoms after rTMS application, but the symptoms were mild and disappeared within 1-2 days. Kohútová et al. [18] noted that TBS was tolerated well with mild side effects, primarily comprising mild and transient headache symptoms. In the two studies of PHN patients [25, 37], there were slight symptoms such as dry mouth, headache, neck pain, and dizziness. Lindholm et al. [36] reported 2 patients who developed unpleasant temporalis contractions. In summary, rTMS in patients with NOP caused only mild discomfort.

## 4. Discussion

As far as we know, there has been no systematic review for rTMS on pain intensity, psychological conditions, and quality of life in individuals with NOP. Overall, the rTMS intervention is safe and has consistent benefits in reducing pain, but improvements in mental health and QOL are difficult to detect, and long-term treatment can have positive effects on QOL. Different study designs and stimulus patterns affect the stability and reliability of the results. The stability and reliability of rTMS treatment in patients with NOP are influenced by study designs and stimulus regimens.

### 4.1. The Mechanisms and Parameters of TMS Action on NOP

rTMS may reduce neuropathic pain in NOP patients by regulating the excitatory activation of the pain circuit in the cerebral cortex, inhibiting the transmission of pain signals through the spinothalamic pathway, and also acting on the neural plasticity of brain regions implicated in the modulation of pain [39]. Studies have demonstrated that high-frequency stimulation (≥5 Hz) delivered to M1 of NOP patients (Evidence Level A) has a definite analgesic effect, suggesting it could be used for treating related diseases [13]. For the treatment of patients with neuropathic pain, stimulation of the motor cortex with rTMS at no less than 1000 pulses at 5-20 Hz can reduce pain intensity by about 25-30% [40]. NOP belongs to chronic peripheral neuropathic pain, and high-frequency rTMS of the M1 region has an obvious analgesic effect, confirming that M1 is the classic region for pain treatment. All factors and mechanisms are ultimately manifested in the activation of the pain network and the production of pain sensation, while rTMS can be regulated in both the upstream and downstream directions and affect the change of the whole pain neural circuit, which also provides a feasible scientific theoretical basis for the treatment of NOP. For all these reasons, the effect of age or pain duration does not appear to be a key factor in treatment effectiveness, so it may be unreasonable to set age or pain duration limits for selecting suitable patients with rTMS. In addition, functional connections between the M1, S1, and S2 and the insular cortex were found to some extent [41]. The effect of S2 stimulation can be explained by its location near the insular cortex, which is important for pain perception. Connections to the S2 are particularly strong during painful stimuli, which may be one reason for mild discomfort [42]. However, stimulation of the S1 was considered to be inefficient or to cause hyperalgesia in some earlier studies [43]. Contrary to the previous view that the best stimulus target may be adjacent areas rather than the corresponding pain “hot spot,” S2 stimulation induces better analgesia regardless of the pain level [44]. Thus, the right S2 appears to be a potential target for NOP treatment.

### 4.2. Effects of rTMS on Psychological Conditions of NOP Patients

NOP will cause different degrees of psychological conditions in patients, and long-term pain will cause them depression, anxiety, and other adverse emotions [45]. The application of rTMS in some brain regions has significant effects on emotional regulation, depression regulation, and other psychological regulations, which may be because the knot structure involved in these regions is intrinsically related. Stimulation of DLPFC targets by rTMS can change the psychological conditions of patients. Conventional rTMS includes high frequency to the left DLPFC and low frequency to the right DLPFC. However, not all depressed patients could benefit from standard rTMS protocols. DLPFC is a key brain region in cognitive and emotional regulation circuits. High-frequency rTMS stimulates the left DLPFC to enhance the activity of neurons in local brain regions, which is a therapeutic principle for rTMS to correct the lateralization of abnormal brain functions. In the study of Umezaki et al. [38], stimulation of the left DLPFC with 10 Hz rTMS did not achieve the expected improvement in psychological conditions. This may be limited by the research protocol. Bares et al. [46] found that stimulation of the right DLPFC region by 1 Hz rTMS was effective in treating refractory depression. Sixty patients with depression who had not previously responded to treatment with one or more antidepressants were randomly assigned to receive 1 Hz rTMS, which showed improvement in depression. The Montgomery-Asberg Depression Rating Scale (MADRS) score improved significantly compared with pretreatment. In the study of Bystritsky et al. [47], it has long been found that stimulation of the right DLPFC by low-frequency rTMS can also reduce generalized anxiety. Ten participants completed six sessions of rTMS over a 3-week period, stereotaxically directed to a previously identified prefrontal location. However, in the study of Umezaki et al. [38], stimulation of the left DLPFC with 10 Hz rTMS did not achieve the expected improvement in psychological conditions. This may be because the choice of stimulus frequency leads to different results. It is also worth exploring whether there is a link with the type of depression.

### 4.3. Effects of rTMS on QOL of NOP Patients

The improvement of QOL in patients with NOP by rTMS may come from two aspects. The reduction of pain makes patients have a great change in their attitude towards life, which enhances their confidence not only physically but also psychologically. QOL covers many aspects, such as sleep quality, work efficiency, and social interaction. The improvement of sleep quality is also the premise for patients to ensure the above. In two studies on PHN patients [25, 37], high-frequency stimulation of the M1 position also improved the sleep quality of patients in the short term. In the only study of BMS [38], these patients experienced daily and deep bilateral burning of the oral mucosa for 4-6 months with no disturbance to appetite or sleep. Umezaki et al. [38] confirmed in their study that high-frequency stimulation of the DLPFC site had positive effects on BMS in terms of pain, psychology, and QOL. Analysis showed that rTMS did not improve the quality of life of NOP patients in the short term by stimulating the left DLPFC site but could slightly improve the quality of life of NOP patients over time. According to the discussion on pain and psychological conditions, the reason why the left DLPFC can improve the quality of life may be caused by the improvement of patients' pain and psychological conditions. The reasons for the failure to improve quality of life may be influenced by the treatment cycle, and the choice of the left DLPFC site may also be potentially associated with the improvement of quality of life.

### 4.4. Limitations

This systematic review was based on only 6 articles, which may have publication bias. The biggest limitation of this study is that no large-scale randomized controlled trials were integrated into the meta-analysis. The sample size of this study was small, and the follow-up time for each study may be too short to determine any long-term treatment regimen resulting in a reduction in positive symptoms. There were many differences in the types and severity of disease among the patients included in the study, so there may be some heterogeneity.

### 4.5. Clinical Application and Prospect

The evidence presented in this review suggests that high-frequency rTMS should be used to improve the clinical symptoms of NOP. NOP, as a common type of neuropathic pain, can be treated clinically with high-frequency rTMS, but the specific site of treatment should also be evaluated based on symptoms. Further research studies should use larger sample sizes and include more patients with NOP-related diseases, such as trigeminal neuralgia. Confirming the parameters and optimal position of coil placement significantly improved the therapeutic effect. This model can be integrated into future studies to standardize rTMS treatment for NOP. Intervention parameters for rTMS should be standardized in addition to blind evaluators, subjects, and therapists. Future emphasis should also be placed on designing RCTs with sufficiently large samples to measure the clinically relevant effects of rTMS on NOP symptoms.

## 5. Conclusion

In summary, high-frequency rTMS is a very safe intervention and may serve as one of the therapeutic modalities to reduce pain intensity in individuals with NOP. There is moderate-to-high evidence to prove that high-frequency rTMS is effective in individuals with NOP, but it has no significant positive effect on psychological conditions and QOL. This study was limited by the number of high-quality studies and the nature of the target population, and more recommendations are needed to encourage further validation in large-sample, multicenter, randomized, double-blind trials.

## Figures and Tables

**Table 1:** Rating of the PEDro scale and level of evidence.

Study criteria	Fricová et al. [35]	Lindholm et al. [36]	Ma et al. [37]	Umezaki et al. [38]	Kohútová et al. [18]	Pei et al. [25]
Eligibility criteria	Yes	Yes	Yes	Yes	Yes	Yes
Random allocation	1	1	1	1	1	1
Concealed allocation	1	1	1	1	1	1
Baseline comparability	1	1	1	1	1	1
Blinded participants	1	0	0	0	1	1
Blinded therapists	1	1	0	1	1	1
Blinded assessors	0	0	0	0	0	0
Adequate follow-up	1	0	1	1	1	0
Intention-to-treat analysis	1	1	1	1	1	1
Between-group comparisons	1	1	1	1	1	1
Point estimates and variability	1	1	1	1	1	1
Total PEDro score	9	7	7	8	9	8
Sample size ≥ 50	Yes	No	No	No	No	Yes
Level of evidence	1	2	2	2	2	1

**Table 2:** Grading of Recommendations Assessment, Development, and Evaluation (GRADE) quality of evidence.

Outcome	Number of studies	Design	Study limitations	Inconsistency	Indirectness	Imprecision	Publication bias	Effect size	GRADE quality	Symbolic expression
Pain intensity: VAS or NRS	6	RCT	0	0	0	0	0	0	High	⨁⨁⨁⨁
Psychological conditions: BDI or SDS	4	RCT	0	0	0	-1^∗^	0	0	Moderate	⨁⨁⨁⊖
Quality of life: QOL or SQ	3	RCT	0	0	0	-1^∗^	0	0	Moderate	⨁⨁⨁⊖
Sensory testing: QST	2	RCT	0	0	0	-1^∗^	0	0	Moderate	⨁⨁⨁⊖

Note: BDI: Beck Depression Inventory; VAS: Visual Analogue Scale; SDS: Self-rating Depression Scale; SQ: sleep quality; QST: quantitative sensory testing; RCT: randomized controlled trial. ^∗^Downgraded by levels due to small sample size.

**Table 3:** Characteristics of participants with NOP in the reviewed studies.

Study	Study design	Duration	NOP condition	Inclusion criteria	Exclusion criteria	Adverse events
Fricová et al. [35]	A double-blind placebo-controlled trial	At least 6 months	Chronic orofacial pain (trigeminal neuralgia, atypical orofacial pain, postherpetic neuralgia, dental pain)	(a) Orofacial pain syndrome, intractable pharmacoresistant pain (b) Stable analgesic medication for at least 1 month before the start of the study and throughout its course and during follow-up evaluation two weeks after completion of rTMS (c) 18-65 years of age	(a) Severe organic brain damage or other serious diseases (b) Which could interfere with rTMS (epilepsy) (c) Any metallic implants in the body (restrictions similar to those for an MRI)	No

Lindholm et al. [36]	A randomized placebo-controlled crossover study	2-30 years	Chronic drug-resistant neuropathic orofacial pain (trigeminal neuropathic pain, atypical facial pain, burning mouth syndrome)	(a) Chronic daily neuropathic pain 4 in severity using NRS of 0 to 10 (b) Patients had no history of seizure, pacemaker implantation, major stroke, or other contraindication for TMS	(a) Multiple ischemic lesions and another after the pain diary follow-up because of average pain less than 4 on the NRS (b) Major depression	Unpleasant temporalis contractions

Ma et al. [37]	A sham stimulation-controlled randomized trial	Longer than 6 months	Postherpetic neuralgia	(a) Patients with chronic pain, moderate to severe in intensity (VAS ≥ 4) despite optimized pharmacological treatment (b) Pain lasting longer than 1 month	(a) Inability to participate in the questionnaires (b) The presence of suicidal ideation and the presence of contraindications for rTMS	Slight dry mouth, headache, neck pain, and dizziness symptoms

Umezaki et al. [38]	A randomized controlled single-blind study	63.42 ± 65.51 months	Burning mouth syndrome	(a) Diagnosed as having BMS daily and deep bilateral burning sensation of the oral mucosa (b) Burning sensation for at least 4-6 months, constant intensity or increasing intensity during the day	(a) Inflammation or autoimmune disease (b) Major depression or major personality disorders or a history of substance abuse (except caffeine or nicotine)	Slight headache

Kohútová et al. [18]	A double-blind sham-controlled parallel-group randomized study	At least 6 months	Chronic orofacial pain	(a) Orofacial pain syndrome in the duration of at least 6 months, intractable pharmacotherapy-resistant pain (b) 18-65 years of age	(a) Severe organic brain damage or other serious diseases	Comprising mild and transient headache symptoms

Pei et al. [25]	A double-blind sham-controlled randomized trial	Lasting for over 1 month	Postherpetic neuralgia	(a) Aged above 50 years old (b) Conforming to the diagnostic criteria of PHN, PHN lasting for over one month. VAS above 4, and having clear consciousness	(a) Personal or family history of epilepsy (b) History of craniocerebral surgery (c) Intracranial implants (d) Cardiac pacemakers (e) Heart, liver, or kidney insufficiency and coagulation disorders	Slight dry mouth, headache, neck pain, and dizziness symptoms

**Table 4:** The main characteristics of rTMS in the reviewed studies.

Study	Study design	Main diagnosis	EG									CG									Measurement
			*n*	Female/male (*n*)	Stimulation site	Age (yrs)	Frequency (Hz)	MT	ITI (s)	Coil type	Total pulses	*n*	Female/male (*n*)	Stimulation site	Age (yrs)	Frequency (Hz)	MT	ITI (s)	Coil type	Total pulses	
Fricová et al. [35]	A double-blind placebo-controlled trial	COP	23	16/7	N/A	50.7	20	95% MT	1.9	N/A	720	36	N/A	N/A	33-65 yrs	10/0	95% MT	1.9	N/A	720	VAS QST
Lindholm et al. [36]	A randomized placebo-controlled crossover study	COP	10	N/A	S2	39-74	10	90% RMT	10	Figure-of-eight coil	1000	6	N/A	M1/S1	39-74	10	90% RMT	10	Figure-of-eight coil	1000	NRS BDI QOL SF-36 BPI NePIQoL
Ma et al. [37]	A sham stimulation-controlled randomized trial	PHN	20	9/11	M1	65.4 ± 10.5	10	80% RMT	3	Round coil	1500	20	11/9	M1	67.3 ± 11.9	10	80% RMT	3	Round coil	1500	VAS SF-MPQ QOL SQ PGIC SDS
Umezaki et al. [38]	A randomized controlled single-blind study	BMS	12	N/A	L-DLPFC	63.36 ± 10.78	10	110% RMT	10	Figure-of-eight coil	3000	8	N/A	L-DLPFC	64.42 ± 8.35	10	110% RMT	10	Figure-of-eight coil	3000	VAS SF-MPQ BPI PHQ-9 PGIC CGI
Kohútová et al. [18]	A double-blind sham-controlled parallel-group randomized study	COP	10	6/4	M1	55.5 ± 12.7	50	90% MT	8	N/A	600	9	6/3	M1	59.3 ± 14.9	50	90% MT	10	N/A	600	VAS BDI BAI QST
Pei et al. [25]	A double-blind sham-controlled randomized trial	PHN	40	21/19	M1	65.9 ± 12.3/65.4 ± 10.5	5/10	80% MT	2.5	N/A	1500	20	9/11	M1	67.3 ± 11.9	0	80% MT	3	N/A	1500	V AS SF-MPQ QOL SQ SDS PGIC

Note: BMS: burning mouth syndrome; BPI: Brief Pain Inventory; BDI: Brief Depression Inventory; CG: control group; COP: chronic orofacial pain; CGI-I: Clinical Global Impression for global improvement scale; EG: experimental group; iTBS: intermittent theta burst stimulation; imTBS: intermediate theta burst stimulation; ITI: intertrain interval; L-DLPFC: left dorsolateral prefrontal cortex; M1/S1/S2: primary motor cortex (M1), primary sensory cortex (S1), and secondary somatosensory cortex (S2); NePIQoL: neuropathic pain impact on quality of life; MT: motor threshold; N/A: not available; NRS: Numerical Rating Scales; PHN: postherpetic neuralgia; PGIC: Patients' Global Impression of Change; PHQ-9: Patient Health Questionnaire; QOL: quality of life; QST: quantitative sensory testing; RMT: resting active motor threshold; SDS: Self-rating Depression Scale; SF-MPQ: Short-Form McGill Pain Questionnaire; SQ: sleep quality; VAS: Visual Analogue Scale.

## Data Availability

The data used to support the finding of this study are available from the corresponding authors upon request.
